# 1-(Furan-2-yl)-2-(2*H*-indazol-2-yl)ethanone

**DOI:** 10.1107/S1600536814006606

**Published:** 2014-03-29

**Authors:** Özden Özel Güven, Gökhan Türk, Philip D. F. Adler, Simon J. Coles, Tuncer Hökelek

**Affiliations:** a Department of Chemistry, Bülent Ecevit University, 67100 Zonguldak, Turkey; b Department of Chemistry, Southampton University, SO17 1BJ Southampton, England; c Department of Physics, Hacettepe University, 06800 Beytepe, Ankara, Turkey

## Abstract

The asymmetric unit of the title compound, C_13_H_10_N_2_O_2_, contains two crystallographically independent mol­ecules (*A* and *B*). The indazole ring systems are approximately planar [maximum deviations = 0.0037 (15) and −0.0198 (15) Å], and their mean planes are oriented at 80.10 (5) and 65.97 (4)° with respect to the furan rings in mol­ecules *A* and *B*, respectively. In the crystal, pairs of C—H⋯N hydrogen bonds link the *B* mol­ecules, forming inversion dimers. These dimers are bridged by the *A* mol­ecules *via* C—H⋯O hydrogen bonds, forming sheets parallel to (011). There are also C—H⋯π inter­actions present, and π–π inter­actions between neighbouring furan and the indazole rings [centroid–centroid distance = 3.8708 (9) Å] of inversion-related mol­ecules, forming a three-dimensional structure.

## Related literature   

For related structures, see: Peeters *et al.* (1979 ▶); Freer *et al.* (1986 ▶); Özel Güven *et al.* (2008*a* ▶,*b* ▶, 2013 ▶, 2014 ▶).
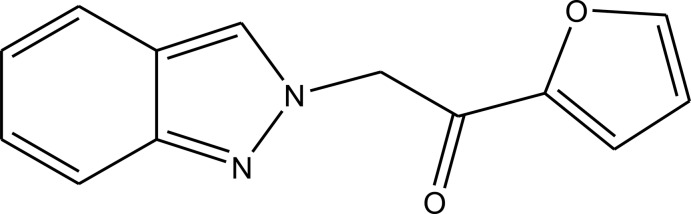


## Experimental   

### 

#### Crystal data  

C_13_H_10_N_2_O_2_
*M* *_r_* = 226.23
Triclinic, 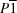

*a* = 9.2899 (3) Å
*b* = 10.6863 (4) Å
*c* = 11.7826 (5) Å
α = 77.046 (3)°
β = 70.780 (3)°
γ = 88.930 (4)°
*V* = 1074.47 (7) Å^3^
*Z* = 4
Mo *K*α radiation
μ = 0.10 mm^−1^
*T* = 294 K
0.17 × 0.15 × 0.10 mm

#### Data collection  

Rigaku Saturn724+ diffractometer
Absorption correction: multi-scan (*CrystalClear*; Rigaku, 2011 ▶) *T*_min_ = 0.984, *T*_max_ = 0.990
10517 measured reflections
5256 independent reflections
4130 reflections with *I* > 2σ(*I*)
*R*_int_ = 0.030

#### Refinement  

*R*[*F* ^2^ > 2σ(*F* ^2^)] = 0.044
*wR*(*F* ^2^) = 0.127
*S* = 1.14
5256 reflections
307 parameters
H-atom parameters constrained
Δρ_max_ = 0.35 e Å^−3^
Δρ_min_ = −0.33 e Å^−3^

### 

Data collection: *CrystalClear* (Rigaku, 2011 ▶); cell refinement: *CrystalClear*; data reduction: *CrystalClear*; program(s) used to solve structure: *SHELXS97* (Sheldrick, 2008 ▶); program(s) used to refine structure: *SHELXL97* (Sheldrick, 2008 ▶); molecular graphics: *ORTEP-3 for Windows* (Farrugia, 2012 ▶); software used to prepare material for publication: *WinGX* (Farrugia, 2012 ▶) and *PLATON* (Spek, 2009 ▶).

## Supplementary Material

Crystal structure: contains datablock(s) I, global. DOI: 10.1107/S1600536814006606/su2716sup1.cif

Structure factors: contains datablock(s) I. DOI: 10.1107/S1600536814006606/su2716Isup2.hkl

Supporting information file. DOI: 10.1107/S1600536814006606/su2716Isup3.cml

CCDC reference: 993569

Additional supporting information:  crystallographic information; 3D view; checkCIF report

## Figures and Tables

**Table 1:** Hydrogen-bond geometry (Å, °) *Cg*1, *Cg*2, *Cg*5 and *Cg*6 are the centroids of the O2/C2–C5, N1/N2/C7/C8/C13, N1′/N2′/C7′/C8′/C13′, and C8′–C13′ rings, respectively.

*D*—H⋯*A*	*D*—H	H⋯*A*	*D*⋯*A*	*D*—H⋯*A*
C3′—H3′⋯N2′^i^	0.93	2.57	3.4031 (18)	149
C7—H7⋯O1′^ii^	0.93	2.48	3.2544 (17)	141
C10′—H10′⋯O1^iii^	0.93	2.44	3.2719 (17)	148
C7—H7⋯*Cg*5^iv^	0.93	2.97	3.6000 (16)	126
C9—H9⋯*Cg*6^iv^	0.93	2.81	3.4312 (17)	125
C9′—H9′⋯*Cg*2^v^	0.93	2.94	3.6743 (15)	137
C12′—H12′⋯*Cg*1^i^	0.93	2.63	3.4480 (16)	147

Symmetry codes: (i) 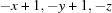; (ii) 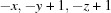; (iii) 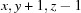; (iv) 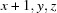; (v) 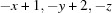.

**Fig. 1.**
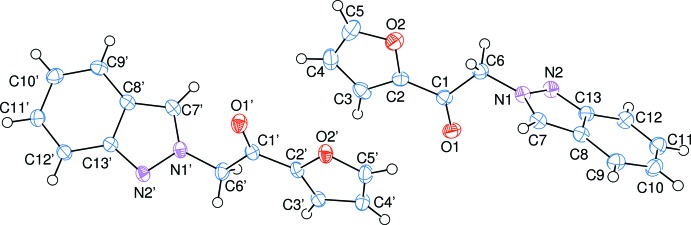
The molecular structure of the two independent molecules (A and B) of the title compound, with atom labelling. Displacement ellipsoids are drawn at the 50% probability level.

**Fig. 2.**
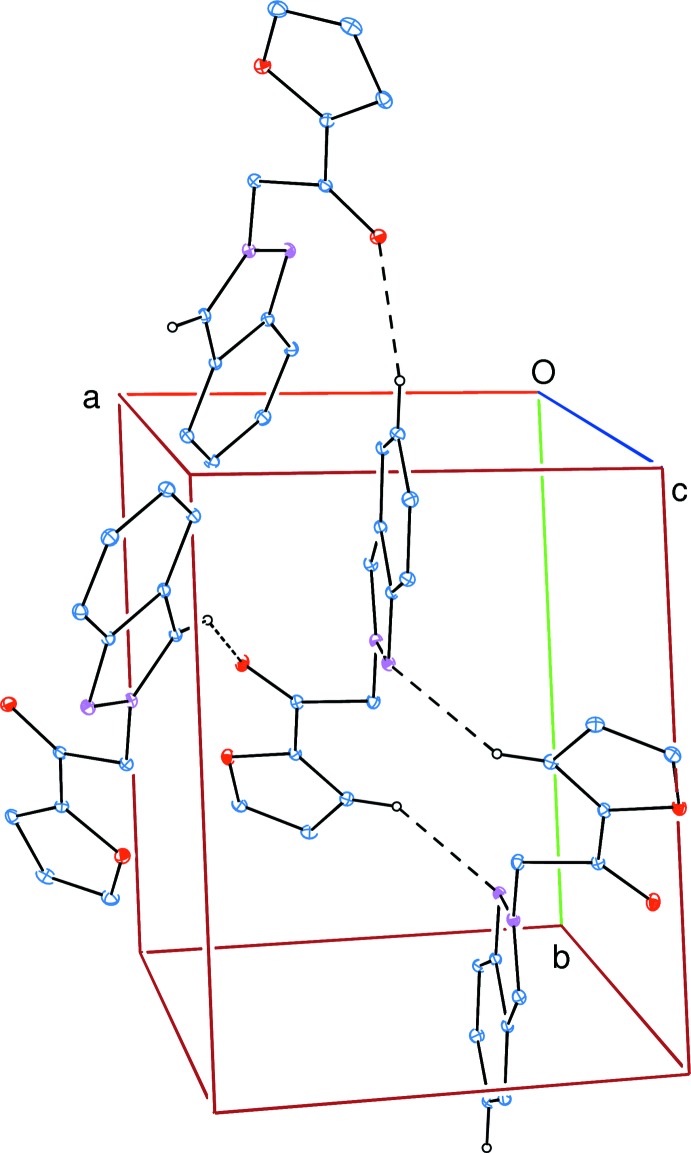
A view of the crystal packing of the title compound with the hydrogen bonds shown as dashed lines [see Table 1 for details; H-atoms not involved in hydrogen bonding have been omitted for clarity].

## supplementary crystallographic information

### 1. Comment

Azole group containing ketones are starting materials of important antifungal compounds like miconazole (Peeters *et al.*, 1979) and econazole (Freer *et al.*, 1986) having an ether structure. The crystal structures of some phenyl ketones having a benzimidazole ring (Özel Güven *et al.*, 2008*a*), a 1,2,4-triazole ring (Özel Güven *et al.*, 2008*b*) and an indazole ring (Özel Güven *et al.*, 2013) have been reported. The crystal structure of indazole ring containing an ether structure has also been reported (Özel Güven *et al.*, 2014). Herein we report on the crystal structure of the title indazole derivative.

The asymmetric unit of the title compound contains two crystallographically independent molecules (A and B), shown in Fig. 1. The bond lengths and angles are generally within normal ranges. The indazole ring systems in the two molecules [N1/N2/C7—C13 and N1'/N2'/C7'—C13'] are approximately planar [maximum deviations of 0.0037 (15) Å for atom C12 and -0.0198 (15) Å for atom C13']. Their mean planes are oriented with respect to the furan rings [O2/C2—C5 and O2'/C2'—C5'] at dihedral angles of 80.10 (5) and 65.97 (4) °, in molecules A and B, respectively. Atoms C6 and C6' are displaced by -0.0508 (14) and 0.1004 (15) Å from their respective indazole ring plane, while atoms O1, C1, C6 and O1', C1', C6' are displaced by 0.0239 (11), -0.0104 (14), -0.1155 (14) and -0.0504 (10), -0.0166 (14), 0.0371 (15) Å from their respective furan ring mean plane.

In the crystal, C—H···N hydrogen bonds link the B molecules forming inversion dimers. These dimers are bridged by the A molecules, via C—H···O hydrogen bonds, forming sheets parallel to (011), see Table 1 and Fig. 2. Weak C—H···π interactions (Table 1) are also present together with π···π interactions between furan and the indazole rings of inversion related molecules Cg1—Cg4^i^, [centroid-centroid distance = 3.8708 (9) Å; symmetry code: (i) - *x+1*, - *y+1*, - *z+1*; Cg1 and Cg4 are the centroids of the rings O2/C2–C5 and N1'/N2'/C7'–C13'] forming a three-dimensional structure.

### 2. Experimental

2-Acetylfuran (3.303 g, 30.01 mmol) was dissolved in 18 ml dioxane/ether (1:2) mixture. This solution was cooled in ice-water, and then bromine (1.96 ml, 38.14 mmol) was added over a period of 30 min.The reaction mixture was stirred for 12 h at room temperature. At the end of this period, saturated ammonium chloride solution (25 ml) was added and the solution extracted with ether. After evaporation of the ether, the residue obtained was purified by column chromatography using a hexane-ethylacetate (60:1) mixture. The resulting furacyl bromide (2.500 g, 13.23 mmol) was dissolved in toluene (30 ml). 1*H*-indazole (3.125 g, 26.45 mmol) was added in small quantities and the reaction mixture was refluxed for 12 h. The solvent was evaporated and the resulting residue was purified by column chromatography using chloroform as eluent. The title ketone was crystallized from 2-propanol to obtain colourless crystals suitable for X-ray analysis [yield 0.9 g; 30%].

### 3. Refinement

H atoms were positioned geometrically with C—H = 0.93 and 0.97 Å for aromatic and methylene H, respectively, and constrained to ride on their parent atoms, with *U*_iso_(H) = 1.2*U*_eq_(C).

### Crystal data

**Table:**

C_13_H_10_N_2_O_2_	*Z* = 4
*M**_r_* = 226.23	*F*(000) = 472
Triclinic, *P*1	*D*_x_ = 1.399 Mg m^−^^3^
Hall symbol: -P 1	Mo *K*α radiation, λ = 0.71073 Å
*a* = 9.2899 (3) Å	Cell parameters from 9141 reflections
*b* = 10.6863 (4) Å	θ = 3.0–28.7°
*c* = 11.7826 (5) Å	µ = 0.10 mm^−^^1^
α = 77.046 (3)°	*T* = 294 K
β = 70.780 (3)°	Block, colourless
γ = 88.930 (4)°	0.17 × 0.15 × 0.10 mm
*V* = 1074.47 (7) Å^3^	

### Data collection

**Table:**

Rigaku Saturn724+ diffractometer	5256 independent reflections
Radiation source: fine-focus sealed tube	4130 reflections with *I* > 2σ(*I*)
Graphite monochromator	*R*_int_ = 0.030
ω scans	θ_max_ = 28.7°, θ_min_ = 3.0°
Absorption correction: multi-scan (*CrystalClear*; Rigaku, 2011)	*h* = −12→11
*T*_min_ = 0.984, *T*_max_ = 0.990	*k* = −14→14
10517 measured reflections	*l* = −15→15

### Refinement

**Table:**

Refinement on *F*^2^	Primary atom site location: structure-invariant direct methods
Least-squares matrix: full	Secondary atom site location: difference Fourier map
*R*[*F*^2^ > 2σ(*F*^2^)] = 0.044	Hydrogen site location: inferred from neighbouring sites
*wR*(*F*^2^) = 0.127	H-atom parameters constrained
*S* = 1.14	*w* = 1/[σ^2^(*F*_o_^2^) + (0.0629*P*)^2^ + 0.1259*P*] where *P* = (*F*_o_^2^ + 2*F*_c_^2^)/3
5256 reflections	(Δ/σ)_max_ < 0.001
307 parameters	Δρ_max_ = 0.35 e Å^−^^3^
0 restraints	Δρ_min_ = −0.33 e Å^−^^3^

### Special details

**Table:**

Geometry. All esds (except the esd in the dihedral angle between two l.s. planes) are estimated using the full covariance matrix. The cell esds are taken into account individually in the estimation of esds in distances, angles and torsion angles; correlations between esds in cell parameters are only used when they are defined by crystal symmetry. An approximate (isotropic) treatment of cell esds is used for estimating esds involving l.s. planes.
Refinement. Refinement of F^2^ against ALL reflections. The weighted R-factor wR and goodness of fit S are based on F^2^, conventional R-factors R are based on F, with F set to zero for negative F^2^. The threshold expression of F^2^ > 2sigma(F^2^) is used only for calculating R-factors(gt) etc. and is not relevant to the choice of reflections for refinement. R-factors based on F^2^ are statistically about twice as large as those based on F, and R- factors based on ALL data will be even larger.

### Fractional atomic coordinates and isotropic or equivalent isotropic displacement parameters (Å^2^)

**Table:**

	*x*	*y*	*z*	*U*_iso_*/*U*_eq_	
O1	0.34655 (12)	0.40513 (9)	0.72286 (9)	0.0301 (2)	
O2	0.12038 (12)	0.66910 (9)	0.67289 (10)	0.0306 (2)	
N1	0.13004 (13)	0.35998 (10)	0.95859 (10)	0.0222 (2)	
N2	0.22341 (13)	0.35403 (11)	1.02685 (10)	0.0240 (3)	
C1	0.24816 (16)	0.48234 (12)	0.74176 (12)	0.0227 (3)	
C2	0.23765 (16)	0.58932 (12)	0.64362 (13)	0.0238 (3)	
C3	0.32561 (18)	0.63034 (14)	0.52231 (13)	0.0295 (3)	
H3	0.4122	0.5936	0.4795	0.035*	
C4	0.2570 (2)	0.74121 (15)	0.47503 (14)	0.0356 (4)	
H4	0.2902	0.7910	0.3945	0.043*	
C5	0.13524 (19)	0.76044 (14)	0.56853 (15)	0.0358 (4)	
H5	0.0703	0.8270	0.5625	0.043*	
C6	0.13153 (16)	0.47746 (12)	0.86879 (12)	0.0236 (3)	
H6A	0.1536	0.5500	0.8986	0.028*	
H6B	0.0307	0.4862	0.8613	0.028*	
C7	0.03917 (15)	0.25281 (12)	0.98598 (12)	0.0228 (3)	
H7	−0.0318	0.2389	0.9492	0.027*	
C8	0.07307 (15)	0.16724 (12)	1.08045 (12)	0.0220 (3)	
C9	0.02026 (16)	0.04070 (13)	1.15061 (13)	0.0267 (3)	
H9	−0.0550	−0.0043	1.1372	0.032*	
C10	0.08333 (17)	−0.01324 (13)	1.23873 (14)	0.0297 (3)	
H10	0.0501	−0.0961	1.2859	0.036*	
C11	0.19855 (18)	0.05475 (14)	1.25960 (14)	0.0302 (3)	
H11	0.2388	0.0151	1.3203	0.036*	
C12	0.25205 (17)	0.17660 (13)	1.19336 (13)	0.0263 (3)	
H12	0.3278	0.2199	1.2079	0.032*	
C13	0.18851 (15)	0.23470 (12)	1.10204 (12)	0.0221 (3)	
O1'	0.12771 (11)	0.65903 (9)	0.22019 (9)	0.0277 (2)	
O2'	0.12290 (11)	0.46552 (9)	0.41588 (8)	0.0256 (2)	
N1'	0.36805 (13)	0.72809 (11)	0.00180 (10)	0.0221 (2)	
N2'	0.36774 (13)	0.71328 (10)	−0.10991 (10)	0.0215 (2)	
C1'	0.23839 (15)	0.59371 (12)	0.21100 (12)	0.0212 (3)	
C2'	0.24799 (15)	0.48921 (12)	0.31045 (11)	0.0210 (3)	
C3'	0.35788 (16)	0.40672 (12)	0.32360 (12)	0.0238 (3)	
H3'	0.4531	0.4032	0.2654	0.029*	
C4'	0.29694 (18)	0.32704 (13)	0.44471 (13)	0.0281 (3)	
H4'	0.3444	0.2603	0.4814	0.034*	
C5'	0.15650 (17)	0.36727 (13)	0.49609 (13)	0.0270 (3)	
H5'	0.0917	0.3321	0.5759	0.032*	
C6'	0.37597 (16)	0.61447 (13)	0.09316 (12)	0.0252 (3)	
H6C	0.4680	0.6225	0.1133	0.030*	
H6D	0.3827	0.5397	0.0582	0.030*	
C7'	0.36543 (16)	0.85057 (13)	0.01246 (12)	0.0238 (3)	
H7'	0.3641	0.8792	0.0817	0.029*	
C8'	0.36502 (15)	0.92660 (12)	−0.10033 (12)	0.0214 (3)	
C9'	0.36551 (15)	1.05980 (13)	−0.15096 (12)	0.0242 (3)	
H9'	0.3624	1.1197	−0.1039	0.029*	
C10'	0.37064 (16)	1.09798 (13)	−0.27098 (13)	0.0264 (3)	
H10'	0.3718	1.1852	−0.3060	0.032*	
C11'	0.37424 (17)	1.00751 (13)	−0.34351 (12)	0.0267 (3)	
H11'	0.3779	1.0372	−0.4249	0.032*	
C12'	0.37248 (16)	0.87798 (12)	−0.29719 (12)	0.0236 (3)	
H12'	0.3747	0.8196	−0.3455	0.028*	
C13'	0.36711 (15)	0.83599 (12)	−0.17304 (12)	0.0201 (3)	

### Atomic displacement parameters (Å^2^)

**Table:**

	*U*^11^	*U*^22^	*U*^33^	*U*^12^	*U*^13^	*U*^23^
O1	0.0299 (6)	0.0234 (5)	0.0321 (6)	0.0032 (4)	−0.0042 (4)	−0.0062 (4)
O2	0.0290 (6)	0.0273 (5)	0.0327 (6)	0.0029 (4)	−0.0084 (4)	−0.0044 (4)
N1	0.0198 (6)	0.0240 (5)	0.0223 (6)	−0.0006 (4)	−0.0066 (4)	−0.0047 (4)
N2	0.0224 (6)	0.0257 (6)	0.0248 (6)	−0.0011 (5)	−0.0091 (5)	−0.0057 (5)
C1	0.0226 (7)	0.0200 (6)	0.0248 (7)	−0.0029 (5)	−0.0065 (5)	−0.0056 (5)
C2	0.0238 (7)	0.0217 (6)	0.0266 (7)	0.0010 (5)	−0.0080 (6)	−0.0076 (5)
C3	0.0294 (8)	0.0318 (7)	0.0267 (7)	−0.0066 (6)	−0.0045 (6)	−0.0118 (6)
C4	0.0491 (10)	0.0320 (8)	0.0268 (8)	−0.0105 (7)	−0.0180 (7)	0.0002 (6)
C5	0.0393 (9)	0.0277 (7)	0.0426 (9)	0.0008 (6)	−0.0216 (8)	−0.0003 (6)
C6	0.0228 (7)	0.0220 (6)	0.0238 (7)	0.0001 (5)	−0.0059 (5)	−0.0038 (5)
C7	0.0186 (6)	0.0255 (7)	0.0242 (7)	−0.0013 (5)	−0.0061 (5)	−0.0068 (5)
C8	0.0184 (6)	0.0239 (6)	0.0225 (6)	−0.0008 (5)	−0.0044 (5)	−0.0065 (5)
C9	0.0232 (7)	0.0247 (7)	0.0297 (7)	−0.0031 (5)	−0.0055 (6)	−0.0058 (5)
C10	0.0306 (8)	0.0229 (7)	0.0304 (7)	−0.0006 (6)	−0.0066 (6)	−0.0011 (5)
C11	0.0320 (8)	0.0302 (7)	0.0293 (8)	0.0051 (6)	−0.0128 (6)	−0.0049 (6)
C12	0.0246 (7)	0.0281 (7)	0.0283 (7)	0.0006 (5)	−0.0113 (6)	−0.0069 (6)
C13	0.0207 (6)	0.0219 (6)	0.0220 (6)	−0.0002 (5)	−0.0049 (5)	−0.0049 (5)
O1'	0.0231 (5)	0.0331 (5)	0.0226 (5)	0.0041 (4)	−0.0050 (4)	−0.0017 (4)
O2'	0.0238 (5)	0.0299 (5)	0.0177 (5)	−0.0005 (4)	−0.0031 (4)	−0.0005 (4)
N1'	0.0214 (6)	0.0248 (6)	0.0168 (5)	−0.0008 (4)	−0.0037 (4)	−0.0021 (4)
N2'	0.0216 (6)	0.0241 (5)	0.0169 (5)	−0.0010 (4)	−0.0044 (4)	−0.0040 (4)
C1'	0.0207 (7)	0.0247 (6)	0.0190 (6)	−0.0004 (5)	−0.0072 (5)	−0.0053 (5)
C2'	0.0213 (6)	0.0244 (6)	0.0157 (6)	−0.0019 (5)	−0.0044 (5)	−0.0039 (5)
C3'	0.0264 (7)	0.0249 (6)	0.0201 (6)	0.0009 (5)	−0.0075 (5)	−0.0057 (5)
C4'	0.0378 (8)	0.0242 (7)	0.0237 (7)	0.0020 (6)	−0.0138 (6)	−0.0025 (5)
C5'	0.0319 (8)	0.0259 (7)	0.0200 (6)	−0.0035 (6)	−0.0083 (6)	0.0008 (5)
C6'	0.0225 (7)	0.0276 (7)	0.0204 (6)	0.0028 (5)	−0.0041 (5)	−0.0004 (5)
C7'	0.0232 (7)	0.0282 (7)	0.0201 (6)	−0.0022 (5)	−0.0058 (5)	−0.0075 (5)
C8'	0.0187 (6)	0.0238 (6)	0.0206 (6)	−0.0016 (5)	−0.0040 (5)	−0.0064 (5)
C9'	0.0219 (7)	0.0242 (6)	0.0255 (7)	−0.0017 (5)	−0.0038 (5)	−0.0099 (5)
C10'	0.0260 (7)	0.0209 (6)	0.0271 (7)	−0.0010 (5)	−0.0040 (6)	−0.0025 (5)
C11'	0.0303 (7)	0.0265 (7)	0.0199 (7)	0.0007 (6)	−0.0061 (6)	−0.0018 (5)
C12'	0.0272 (7)	0.0236 (6)	0.0200 (6)	−0.0008 (5)	−0.0069 (5)	−0.0060 (5)
C13'	0.0180 (6)	0.0210 (6)	0.0198 (6)	−0.0012 (5)	−0.0045 (5)	−0.0045 (5)

### Geometric parameters (Å, º)

**Table:**

O1—C1	1.2190 (17)	O1'—C1'	1.2207 (16)
O2—C2	1.3720 (17)	O2'—C2'	1.3701 (16)
O2—C5	1.3562 (18)	O2'—C5'	1.3528 (16)
N1—N2	1.3556 (15)	N1'—C6'	1.4496 (16)
N1—C6	1.4462 (16)	N1'—C7'	1.3417 (17)
N1—C7	1.3488 (16)	N2'—N1'	1.3614 (15)
N2—C13	1.3550 (16)	N2'—C13'	1.3559 (16)
C1—C2	1.4598 (18)	C1'—C6'	1.5214 (18)
C1—C6	1.5219 (19)	C2'—C1'	1.4514 (18)
C2—C3	1.366 (2)	C2'—C3'	1.3610 (19)
C3—C4	1.425 (2)	C3'—C4'	1.4223 (19)
C3—H3	0.9300	C3'—H3'	0.9300
C4—C5	1.344 (2)	C4'—H4'	0.9300
C4—H4	0.9300	C5'—C4'	1.353 (2)
C5—H5	0.9300	C5'—H5'	0.9300
C6—H6A	0.9700	C6'—H6C	0.9700
C6—H6B	0.9700	C6'—H6D	0.9700
C7—H7	0.9300	C7'—H7'	0.9300
C8—C7	1.3922 (19)	C8'—C7'	1.3948 (18)
C8—C9	1.4174 (18)	C9'—C8'	1.4141 (18)
C9—H9	0.9300	C9'—C10'	1.3660 (19)
C10—C9	1.369 (2)	C9'—H9'	0.9300
C10—C11	1.420 (2)	C10'—C11'	1.4202 (19)
C10—H10	0.9300	C10'—H10'	0.9300
C11—H11	0.9300	C11'—H11'	0.9300
C12—C11	1.3655 (19)	C12'—C11'	1.3675 (18)
C12—H12	0.9300	C12'—C13'	1.4150 (18)
C13—C8	1.4231 (18)	C12'—H12'	0.9300
C13—C12	1.4130 (19)	C13'—C8'	1.4255 (18)
			
C5—O2—C2	106.78 (12)	C5'—O2'—C2'	106.24 (11)
N2—N1—C6	119.17 (10)	N2'—N1'—C6'	118.38 (11)
C7—N1—N2	114.56 (11)	C7'—N1'—N2'	114.33 (11)
C7—N1—C6	126.24 (11)	C7'—N1'—C6'	127.26 (11)
C13—N2—N1	103.11 (10)	C13'—N2'—N1'	102.97 (10)
O1—C1—C2	121.81 (13)	O1'—C1'—C2'	122.77 (12)
O1—C1—C6	122.96 (12)	O1'—C1'—C6'	122.35 (11)
C2—C1—C6	115.20 (12)	C2'—C1'—C6'	114.88 (12)
O2—C2—C1	117.61 (12)	O2'—C2'—C1'	115.92 (12)
C3—C2—O2	109.94 (12)	C3'—C2'—O2'	110.48 (11)
C3—C2—C1	132.44 (14)	C3'—C2'—C1'	133.60 (12)
C2—C3—C4	105.59 (14)	C2'—C3'—C4'	105.77 (13)
C2—C3—H3	127.2	C2'—C3'—H3'	127.1
C4—C3—H3	127.2	C4'—C3'—H3'	127.1
C3—C4—H4	126.4	O2'—C5'—H5'	124.5
C5—C4—C3	107.20 (13)	C4'—C5'—O2'	110.93 (12)
C5—C4—H4	126.4	C4'—C5'—H5'	124.5
O2—C5—H5	124.8	C3'—C4'—H4'	126.7
C4—C5—O2	110.48 (14)	C5'—C4'—C3'	106.58 (13)
C4—C5—H5	124.8	C5'—C4'—H4'	126.7
N1—C6—C1	113.33 (11)	N1'—C6'—C1'	112.61 (11)
N1—C6—H6A	108.9	N1'—C6'—H6C	109.1
N1—C6—H6B	108.9	N1'—C6'—H6D	109.1
C1—C6—H6A	108.9	C1'—C6'—H6C	109.1
C1—C6—H6B	108.9	C1'—C6'—H6D	109.1
H6A—C6—H6B	107.7	H6C—C6'—H6D	107.8
N1—C7—C8	106.09 (11)	N1'—C7'—C8'	106.76 (11)
N1—C7—H7	127.0	N1'—C7'—H7'	126.6
C8—C7—H7	127.0	C8'—C7'—H7'	126.6
C7—C8—C9	135.26 (13)	C7'—C8'—C9'	135.85 (12)
C7—C8—C13	104.46 (11)	C7'—C8'—C13'	103.89 (11)
C9—C8—C13	120.28 (12)	C9'—C8'—C13'	120.25 (12)
C8—C9—H9	121.0	C8'—C9'—H9'	121.0
C10—C9—C8	118.01 (13)	C10'—C9'—C8'	118.06 (12)
C10—C9—H9	121.0	C10'—C9'—H9'	121.0
C9—C10—C11	121.33 (13)	C9'—C10'—C11'	121.52 (12)
C9—C10—H10	119.3	C9'—C10'—H10'	119.2
C11—C10—H10	119.3	C11'—C10'—H10'	119.2
C10—C11—H11	119.0	C10'—C11'—H11'	119.0
C12—C11—C10	122.04 (13)	C12'—C11'—C10'	121.97 (12)
C12—C11—H11	119.0	C12'—C11'—H11'	119.0
C11—C12—C13	117.78 (13)	C11'—C12'—C13'	117.58 (12)
C11—C12—H12	121.1	C11'—C12'—H12'	121.2
C13—C12—H12	121.1	C13'—C12'—H12'	121.2
N2—C13—C8	111.78 (12)	N2'—C13'—C8'	112.04 (11)
N2—C13—C12	127.66 (12)	N2'—C13'—C12'	127.32 (12)
C12—C13—C8	120.56 (12)	C12'—C13'—C8'	120.61 (11)
			
C5—O2—C2—C1	179.45 (12)	C5'—O2'—C2'—C1'	−179.04 (11)
C5—O2—C2—C3	0.22 (15)	C5'—O2'—C2'—C3'	0.50 (14)
C2—O2—C5—C4	0.01 (16)	C2'—O2'—C5'—C4'	−0.84 (15)
C6—N1—N2—C13	177.98 (11)	N2'—N1'—C6'—C1'	−119.87 (13)
C7—N1—N2—C13	0.09 (15)	C7'—N1'—C6'—C1'	62.35 (18)
N2—N1—C6—C1	89.29 (14)	N2'—N1'—C7'—C8'	−0.71 (16)
C7—N1—C6—C1	−93.08 (15)	C6'—N1'—C7'—C8'	177.15 (12)
N2—N1—C7—C8	0.11 (16)	C13'—N2'—N1'—C6'	−177.16 (11)
C6—N1—C7—C8	−177.61 (12)	C13'—N2'—N1'—C7'	0.91 (15)
N1—N2—C13—C8	−0.25 (15)	N1'—N2'—C13'—C8'	−0.76 (14)
N1—N2—C13—C12	−179.97 (14)	N1'—N2'—C13'—C12'	177.36 (13)
O1—C1—C2—O2	178.16 (12)	O1'—C1'—C6'—N1'	7.19 (18)
O1—C1—C2—C3	−2.8 (2)	C2'—C1'—C6'—N1'	−173.78 (11)
C6—C1—C2—O2	−3.81 (17)	O2'—C2'—C1'—O1'	1.42 (19)
C6—C1—C2—C3	175.21 (14)	O2'—C2'—C1'—C6'	−177.60 (11)
O1—C1—C6—N1	−11.17 (18)	C3'—C2'—C1'—O1'	−177.99 (14)
C2—C1—C6—N1	170.83 (11)	C3'—C2'—C1'—C6'	3.0 (2)
O2—C2—C3—C4	−0.35 (15)	O2'—C2'—C3'—C4'	0.00 (15)
C1—C2—C3—C4	−179.43 (14)	C1'—C2'—C3'—C4'	179.43 (14)
C2—C3—C4—C5	0.35 (16)	C2'—C3'—C4'—C5'	−0.50 (15)
C3—C4—C5—O2	−0.22 (17)	O2'—C5'—C4'—C3'	0.85 (16)
C9—C8—C7—N1	179.74 (15)	C9'—C8'—C7'—N1'	−178.62 (15)
C13—C8—C7—N1	−0.25 (15)	C13'—C8'—C7'—N1'	0.19 (15)
C7—C8—C9—C10	−179.87 (16)	C10'—C9'—C8'—C7'	177.57 (15)
C13—C8—C9—C10	0.1 (2)	C10'—C9'—C8'—C13'	−1.1 (2)
C11—C10—C9—C8	−0.2 (2)	C8'—C9'—C10'—C11'	0.5 (2)
C9—C10—C11—C12	0.0 (2)	C9'—C10'—C11'—C12'	0.1 (2)
C13—C12—C11—C10	0.2 (2)	C13'—C12'—C11'—C10'	−0.1 (2)
N2—C13—C8—C7	0.32 (16)	C11'—C12'—C13'—N2'	−178.52 (13)
N2—C13—C8—C9	−179.67 (12)	C11'—C12'—C13'—C8'	−0.5 (2)
C12—C13—C8—C7	−179.94 (13)	N2'—C13'—C8'—C7'	0.38 (15)
C12—C13—C8—C9	0.1 (2)	N2'—C13'—C8'—C9'	179.41 (12)
N2—C13—C12—C11	179.48 (14)	C12'—C13'—C8'—C7'	−177.89 (12)
C8—C13—C12—C11	−0.2 (2)	C12'—C13'—C8'—C9'	1.2 (2)

### Hydrogen-bond geometry (Å, º)

Cg1, Cg2, Cg5 and Cg6 are the centroids of the O2/C2–C5, N1/N2/C7/C8/C13, N1'/N2'/C7'/C8'/C13', and C8'–C13' rings, respectively.

**Table:**

*D*—H···*A*	*D*—H	H···*A*	*D*···*A*	*D*—H···*A*
C3′—H3′···N2′^i^	0.93	2.57	3.4031 (18)	149
C7—H7···O1′^ii^	0.93	2.48	3.2544 (17)	141
C10′—H10′···O1^iii^	0.93	2.44	3.2719 (17)	148
C7—H7···*Cg*5^iv^	0.93	2.97	3.6000 (16)	126
C9—H9···*Cg*6^iv^	0.93	2.81	3.4312 (17)	125
C9′—H9′···*Cg*2^v^	0.93	2.94	3.6743 (15)	137
C12′—H12′···*Cg*1^i^	0.93	2.63	3.4480 (16)	147

Symmetry codes: (i) −*x*+1, −*y*+1, −*z*; (ii) −*x*, −*y*+1, −*z*+1; (iii) *x*, *y*+1, *z*−1; (iv) *x*+1, *y*, *z*; (v) −*x*+1, −*y*+2, −*z*.

## References

[bb1] Farrugia, L. J. (2012). *J. Appl. Cryst.* **45**, 849–854.

[bb2] Freer, A. A., Pearson, A. & Salole, E. G. (1986). *Acta Cryst.* C**42**, 1350–1352.

[bb3] Özel Güven, Ö., Erdoğan, T., Coles, S. J. & Hökelek, T. (2008*a*). *Acta Cryst.* E**64**, o1358. 10.1107/S1600536808019107 PMC2961837 21202976

[bb4] Özel Güven, Ö., Tahtacı, H., Coles, S. J. & Hökelek, T. (2008*b*). *Acta Cryst.* E**64**, o1604. 10.1107/S1600536808023258 PMC2962216 21203297

[bb5] Özel Güven, Ö., Türk, G., Adler, P. D. F., Coles, S. J. & Hökelek, T. (2013). *Acta Cryst.* E**69**, o184. 10.1107/S1600536812051811 PMC3569246 23424469

[bb6] Özel Güven, Ö., Türk, G., Adler, P. D. F., Coles, S. J. & Hökelek, T. (2014). *Acta Cryst.* E**70**, o410. 10.1107/S1600536814004887 PMC3998539 24826125

[bb7] Peeters, O. M., Blaton, N. M. & De Ranter, C. J. (1979). *Acta Cryst.* B**35**, 2461–2464.

[bb8] Rigaku (2011). *CrystalClear* Rigaku Corporation, Tokyo, Japan.

[bb9] Sheldrick, G. M. (2008). *Acta Cryst.* A**64**, 112–122. 10.1107/S0108767307043930 18156677

[bb10] Spek, A. L. (2009). *Acta Cryst.* D**65**, 148–155. 10.1107/S090744490804362X PMC2631630 19171970

